# Cationic nickel metal-organic frameworks for adsorption of negatively charged dye molecules

**DOI:** 10.1016/j.dib.2018.04.062

**Published:** 2018-04-24

**Authors:** Chizoba I. Ezugwu, Md. Ali Asraf, Xiao Li, Shengwei Liu, Chih-Ming Kao, Serge Zhuiykov, Francis Verpoort

**Affiliations:** a School of Environmental Science and Engineering, Guangdong Provincial Key Laboratory of Environmental Pollution Control and Remediation Technology, Sun Yat-sen University, Guangzhou 510006, PR China; b Laboratory of Organometallics, Catalysis and Ordered Materials, State Key Laboratory of Advanced Technology for Materials Synthesis and Processing, Wuhan University of Technology, Wuhan 430070, PR China; c National Research Tomsk Polytechnic University, Lenin Avenue 30, 634050 Tomsk, Russian Federation; d Ghent University Global Campus, Songdo, 119 Songdomunhwa-Ro, Yeonsu-Gu, Incheon, Republic of Korea; e Institute of Environmental Engineering, National Sun Yat-Sen University, Kaohsiung 80424, Taiwan; f Department of Chemistry, Rajshahi University, Rajshahi 6205, Bangladesh

## Abstract

Industrial dye effluents with low biodegradability are highly toxic and carcinogenic on both human and aquatic lives, thus they are detrimental to the biodiversity of environment. Herein, this data set presents the potential of cationic Nickel based MOFs in the adsorption of charged and neutral dye molecules. Data set include a concise description of experimental conditions for the synthesis of imidazolium ligands, 1,3-bis(4-carboxyphenyl)imidazolium chloride (H_2_L^+^Cl^−^) and 1,3-bis(3,5-dicarboxyphenyl)imidazolium chloride (H_4_L^+^Cl^−^), and MOFs. The data show that the two Nickel MOFs, **1** and **2**, synthesized from imidazolium ligands are cationic frameworks. The adsorption and analysis data show that the cationic MOFs exhibit efficient adsorptive removal capacity for positively charged dyes, adsorbing up to 81.08% and 98.65% of Methyl orange and Congo red, respectively.

**Specifications table**

**Table:** 

**Subject area**	*Chemistry, Environmental Sciences and Engineering*
**More specific subject area**	*Adsorption*
**Type of data**	*Table, image, graph, figure*
**How data was acquired**	– Structural determination of ligands: ^1^H and ^13^C-NMR spectra recorded at 500 and 100 MHz respectively, with Bruker 500 MHz NMR spectrometer – Presence and distribution of elements: Elemental mapping using EDS spectroscopy (EDS, Oxford Instruments, Britain) – Porosity and Surface area measurement of azolium-MOFs: BET and Langmuir surface areas were determined by a volumetric method on a Micrometrics instrument (ASAP2020). – Dye concentration measurement: Monitored by UV–vis absorption spectroscopy(UV-3600, Shimadzu, Japan)
**Data format**	*Analyzed*
**Experimental factors**	*Activation of cationic-MOFs prior to the BETs measurements were achieved by evacuating at 180 °C under vacuum for about 12 h.*
**Experimental features**	– *Azolium ligands were synthesized as reported in the original article [“Submitted to Journal of Colloid and Interface Science.”] and literature procedures* [1], [2] *and then mixed with appropriate amount of nickel nitrate in DMF. The mixture was solvothermally heated to obtain the cationic MOFs.* – *The adsorbent MOFs were mixed with charged organic dye molecules in aqueous media and monitored for their adsorption capacity.*
**Data source location**	*Guangzhou, Wuhan, PR China*
**Data accessibility**	*Data are accessible with article*
**Related research article**	*C. I. Ezugwu, Md. A. Asraf, X. Li, S. Liu, CM. Kao, S. Zhuiykov, F. Verpoort, Selective and adsorptive removal of anionic dyes and CO*_*2*_*with azolium-based metal-organic frameworks “Submitted to Journal of Colloid and Interface Science.”*

**Value of the data**

• The illustrated synthetic route can inspire researchers to design and immobilize other charged moieties in metal-organic frameworks.
• The as-synthesized cationic nickel MOFs has a good potential application for adsorptive removal of negatively charged organic pollutants from contaminated environment.
• The acquired percentage adsorption data will be useful to scientific community intending to investigate further on the nature of the electrochemical interactions between azolium moieties and other charged and neutral organic molecules.

## 1 Data

**Fig. 1:**
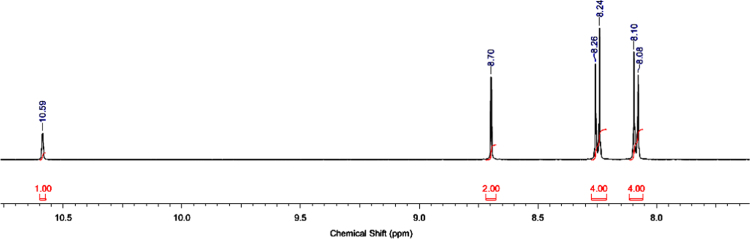
^1^H-NMR spectrum of H_2_L^+^Cl^−^.

**Fig. 2:**
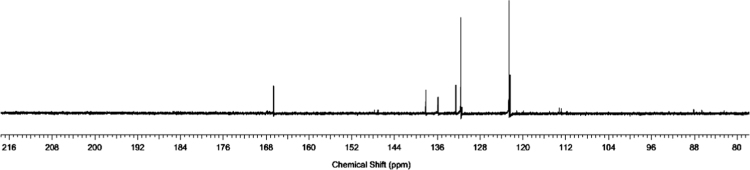
^13^C-NMR spectrum of H_2_L^+^Cl^−^.

**Fig. 3:**
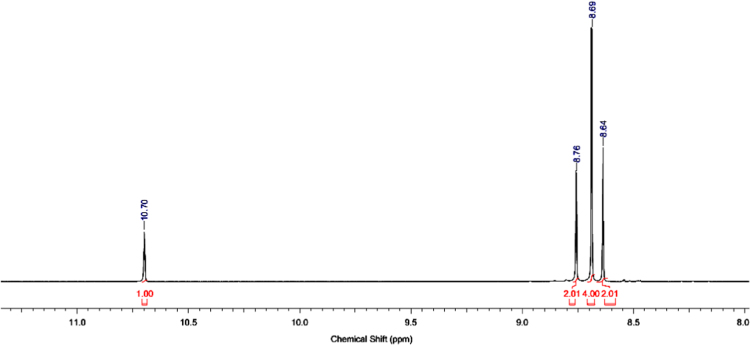
^1^H-NMR spectrum of H_4_L^+^Cl^−^.

**Fig. 4:**
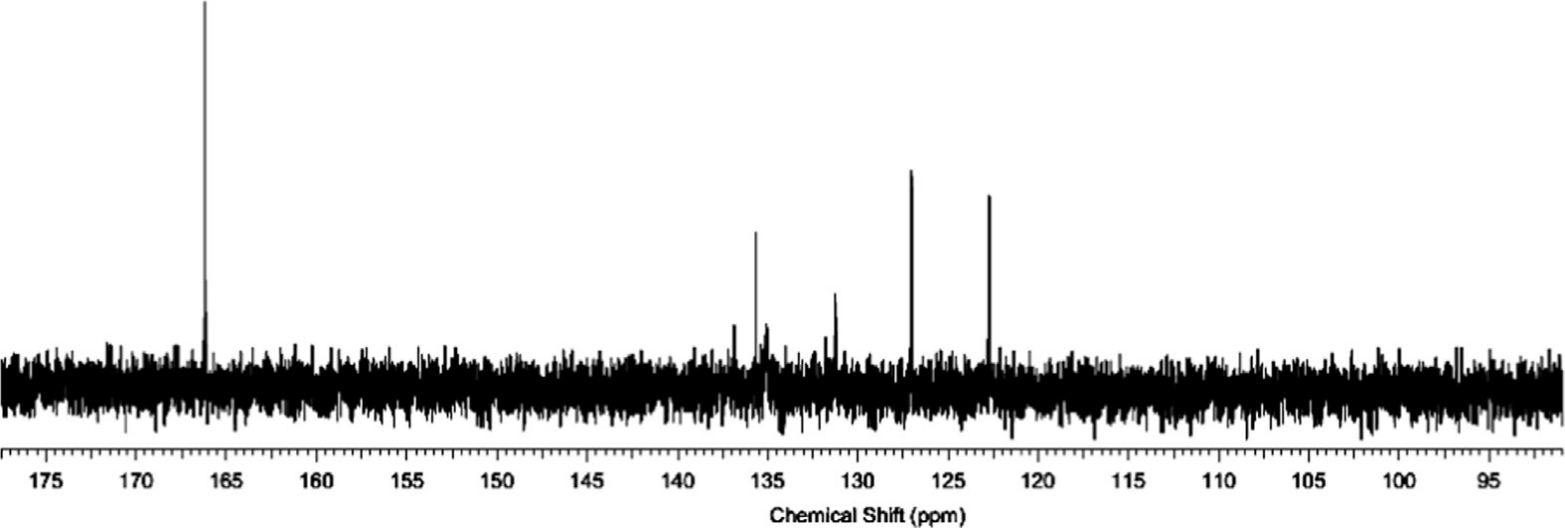
^13^C-NMR spectrum of H_4_L^+^Cl^−^.

The ^1^H and ^13^C NMR spectra for the synthesized azolium ligands, 1,3-bis(4-carboxyphenyl)imidazolium chloride (H_2_L^+^Cl^−^), are presented in Fig. 1, Fig. 2, respectively. Similarly, represented in Fig. 3, Fig. 4 are the ^1^H and ^13^C NMR spectra for 1,3-bis(3,5-dicarboxyphenyl)imidazolium chloride (H_4_L^+^Cl^−^). ^1^H and ^13^C-NMR spectra were recorded at 500 MHz and 100 MHz, respectively and the integration of the peaks in these spectra showed that the two ligands were formed without impurity. The data for ^1^H-NMR spectra for the two ligands showed the characteristic signal of imidazolium proton in the upfield region at 10.59 and 10.70 ppm, confirming the successful formation of the imidazolium ligands.

**Fig. 5:**
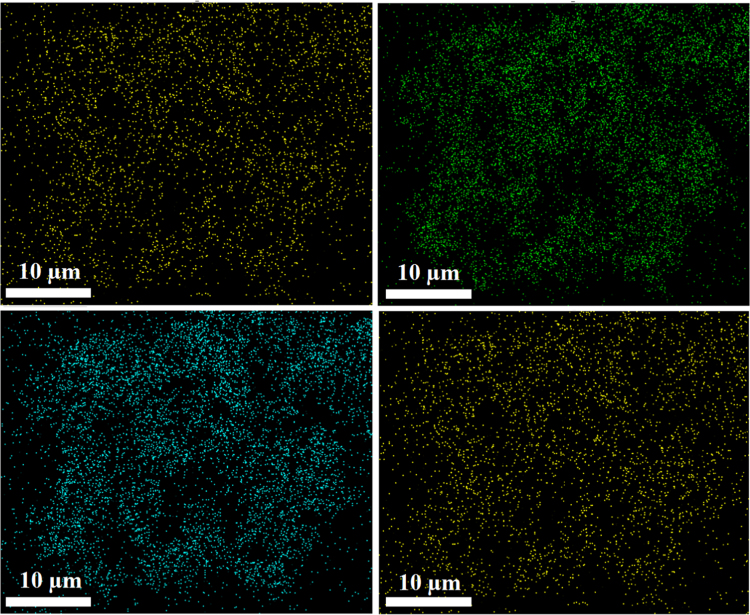
EDS mapping of **2** showing the presence of Ni, C, O and N.

**Scheme 1:**
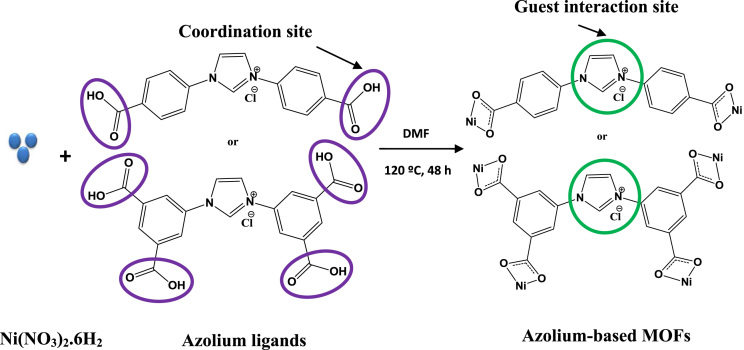
Structural representation of the synthetic route of azolium-based MOFs.

Schematic overview of the structural and synthetic procedure for the two as-prepared cationic nickel based MOFs is concisely illustrated in Scheme 1. The guest interaction sites, which are the positively charged imidazolium components of the frameworks, are highlighted using green circles. Fig. 5 is the EDS mapping of **2,** illustrating that the MOF is comprises of evenly distributed Ni, C, O and N. The Brunauer–Emmett–Teller (BET) and the Langmuir surface areas of the two MOFs are presented in Table 1. Similar BET behavior has been reported for azolium MOFs which can be attributed to high level of interpenetration in the frameworks caused by the shape of the ligand [2], [3].

**Table 1:** BET surface area of 1 and 2.

**MOFs**	**BET Surface Area (m**^**2**^**/g)**	**Langmuir Surface Area (m**^**2**^**/g)**	**Single point total pore volume at *P*/P0 = 0.993625502: (cm**^**2**^**/g)**
**1**	101.66	108.76	0.075
**2**	110.80	168.21	0.090

**Fig. 6:**
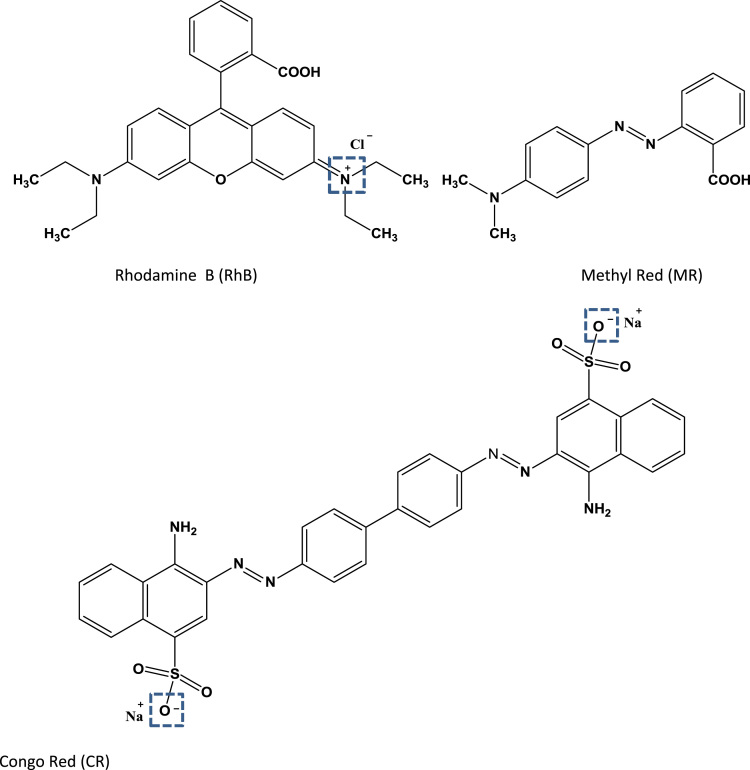
Chemical structures of cationic (MB), neutral (MR) and anionic (CR) dyes with marked electrical sites.

**Fig. 7:**
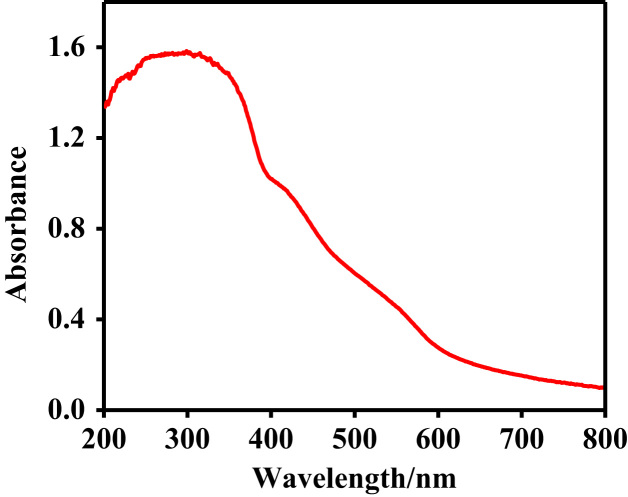
The UV/Vis spectrum of an azolium ligand, 1,3-bis(4-carboxyphenyl)imidazolium chloride.

**Fig. 8:**
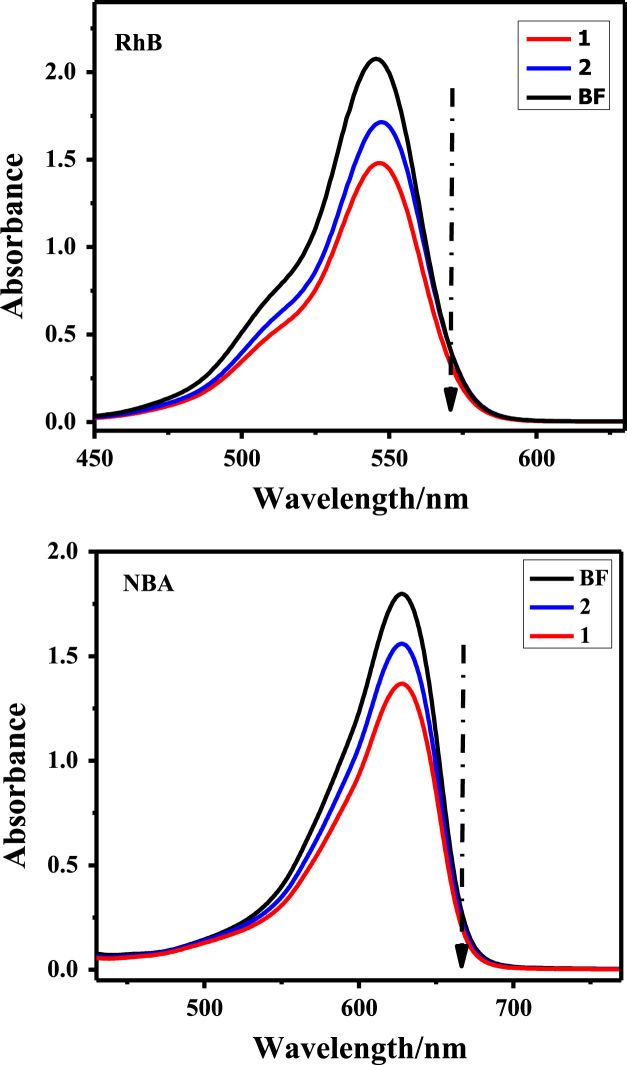
Cationic dyes (RhB & NBA) uptake by MOFs, **1** and **2**, in ethanolic medium. (BF = before MOF addition).

**Fig. 9:**
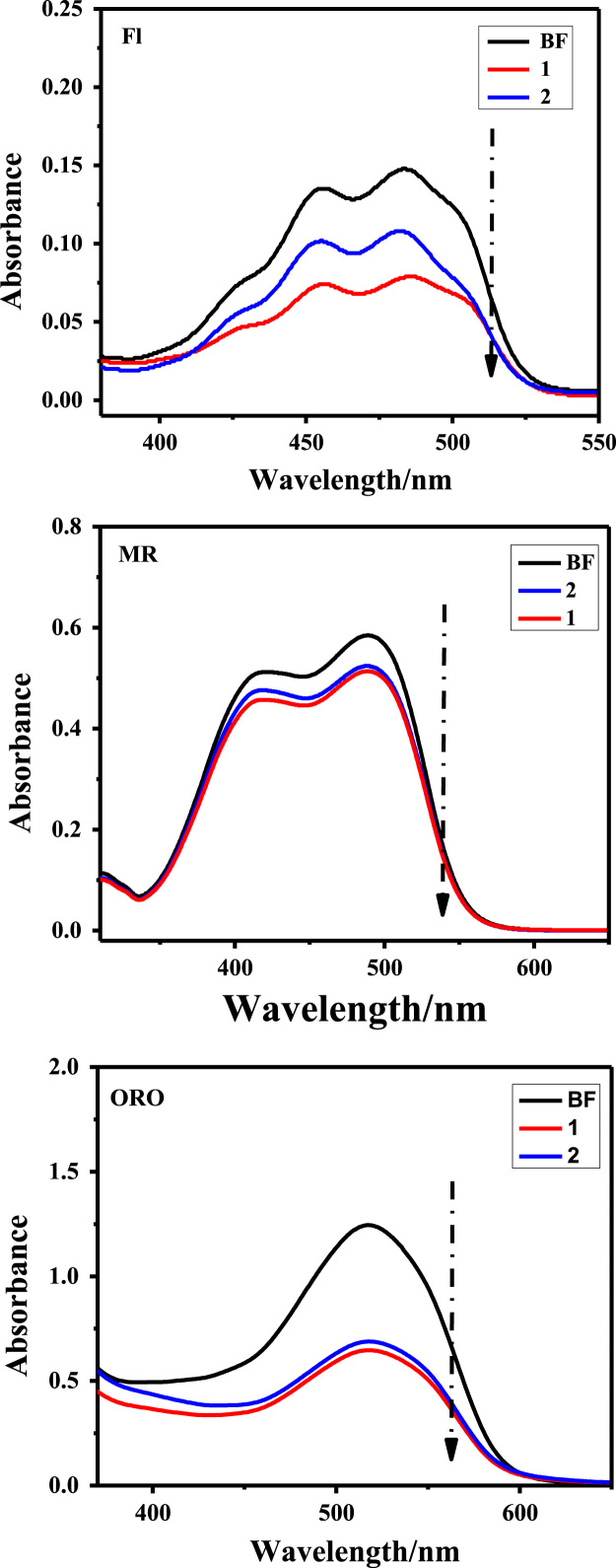
Neutral dyes (Fl, MR & ORO) uptake by MOFs, **1** and **2**, in ethanolic medium. (BF = before MOF addition).

**Fig. 10:**
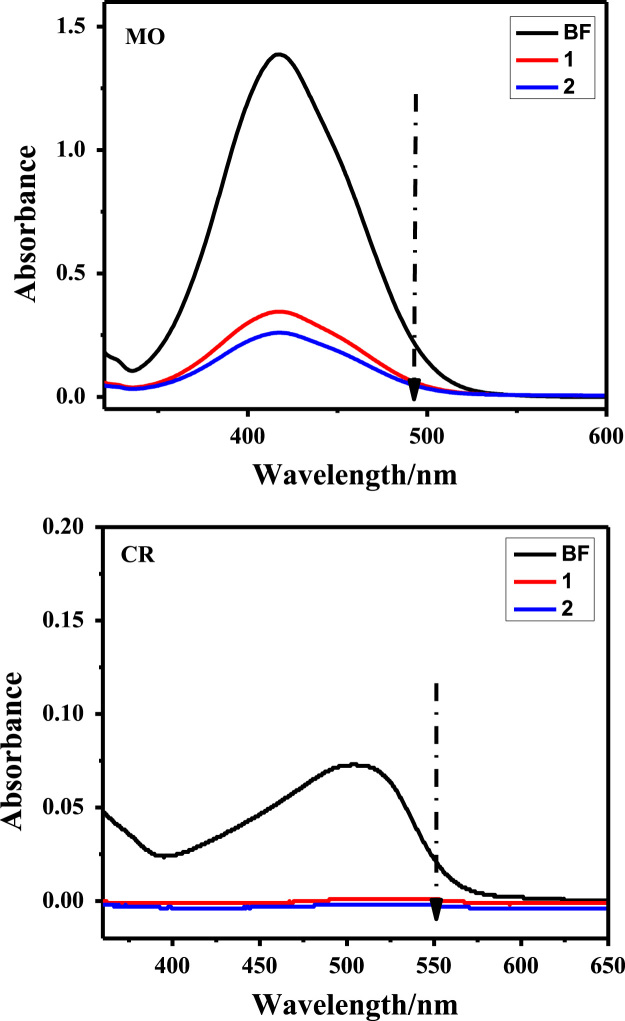
Anionic dyes (MO & CR) uptake by MOFs, **1** and **2**, in ethanolic medium. (BF = before MOF addition).

Owing to the harmful effects of industrial dye effluents to human and entire environment [4], [5], [6], the data sets below illustrate the adsorption potentials of the as-synthesized cationic MOFs on positive, neutral and negative dye molecules. Fig. 6 presents the structures of cationic (RhB), neutral (MR) and anionic (CR) dyes, showing the electrical site as highlighted. The UV–vis spectroscopy of the ligand H_2_L^+^Cl^−^ is represented in Fig. 7. The data on the dye adsorption capacity of the two cationic azolium MOFs are illustrated in Fig. 8, Fig. 9, Fig. 10. Comparatively, the percentage adsorption capacity of MOFs, **1** and **2**, for anionic dyes (97.30%and 98.65% for CR) are much higher than neutral (48.16% and 44.88% for ORO) and cationic (28.69% and 17.41% for RhB) dyes. The higher adsorptive removal of cationic dyes is attributed to the electrostatic interactions between the positive imidazolium moieties (at the guest interaction site, Scheme 1) of the framework and the negative component of the cationic dye, Fig. 6.

## 2 Experimental design, materials, and methods

### 2.1 Materials

All starting materials and solvents were obtained from commercial sources and used without further purification. The synthesis of the ligands and MOFs are detailed in the original paper, [*“Submitted to Journal of Colloid and Interface Science.”]* and are concisely discussed below.

### 2.2 Synthesis of Ligands, 1,3-bis(4-carboxyphenyl)imidazolium chloride (H_2_L^+^Cl¯) and 1,3-bis(3,5-dicarboxyphenyl)imidazolium chloride (H_4_L^+^Cl¯)

The imidazolium ligands were synthesized following the reported procedures with little modification [1], [2]. Briefly, 5 g, 16.89 mmol, of *N*,*N*′-bis(4-carboxyphenyl)ethylenediimine in 30 mL THF was mixed with a solution of paraformaldehyde (635 mg, 21.16 mmol) in 2.1 mL 12 M HCl in 4 mL dioxane at 0 °C. The mixture was stirred for 4 h at room temperature, filtered, washed with Et_2_O and dried in Vacuum. Similar synthetic procedures were employed in the preparation of H_4_L^+^Cl^−^ except that *N*,*N*′-bis(4-carboxyphenyl)ethylenediimine was replaced by *N*,*N*′-bis (3,5-dicarboxyphenyl)ethylenediimine.

### 2.3 Synthesis of MOFs, 1 and 2

The synthesis of MOF, 1, was achieved following literature procedures [1] with slight modification.

Ni(NO_3_)_2_.6H_2_O (290.79 mg, 1.0 mmol) in 3 mL pre-dried DMF was mixed with H_2_L^+^Cl^−^ (86.19 mg, 0.25 mmol) in a Teflon-lined autoclave. The reaction mixture was heated to 120 °C for 48 h and cooled at the rate of 10 °C/h to room temperature. The product was filtered and washed with pre-dried DMF.

Similar procedures were used in the synthesis of MOF, 2, except that the ligand H_2_L^+^Cl^−^ was replaced by H_4_L^+^Cl^−^.

### 2.4 Adsorption experiments

The adsorption of the cationic, anionic and neutral dye molecules by MOFs, **1** and **2** was carried out as follows: 10 mg of as-synthesized MOFs was added to 10 mL aqueous solution of dye (100 mg/L) under stirring and the solution was further stirred for 24 h at room temperature. The mixture was centrifuged and the plasma was analyzed by UV–vis absorption spectroscopy after the solution was diluted to 1/10 of the stock solution.

## 3 Data analysis

The efficiency of the MOFs to adsorb dye molecules is calculated based on percentage of degradation obtained according to Eq. (1).

(1) $$D=\left(A_{0}\;-\;A\right)/\left(A_{0}\right)\times 100\%$$

where; *D* is percentage of degradation (%), *A*_0_ is initial absorbance and *A* is final absorbance after degradation.
