# Supplementary material for: Cationic nickel metal-organic frameworks for adsorption of negatively charged dye molecules
Source: Data Brief. 2018 Apr 24;18:1952–61. doi: 10.1016/j.dib.2018.04.062 (PMC5998174; doi:10.1016/j.dib.2018.04.062)
Supplement: Supplementary file 1 — Supplementary material [file mmc1.docx]

**Conflict of interest**

All the author confirms as No conflict of Interest.
